# Farmer participatory evaluation of *Amaranthus cruentus L.* breeding lines for marketable vegetable yield and organoleptic quality under on-farm and on-station conditions

**DOI:** 10.3389/fpls.2026.1742591

**Published:** 2026-05-11

**Authors:** Ayubu Edward Shimikilo, Fekadu Fufa Dinssa, Roland Schafleitner, Pavithravani Venkataramana

**Affiliations:** 1 School of Life Sciences and Bioengineering (LiSBE), The Nelson Mandela African Institution of Science and Technology, Arusha, Tanzania; 2 World Vegetable Center Eastern and Southern Africa, Arusha, Tanzania; 3 World Vegetable Center, International Maize and Wheat Improvement Center (CIMMYT) Global Headquarters, Texcoco, Mexico

**Keywords:** amaranth, breeding lines, cluster analysis, G × E, gender-disaggregated participatory approach, GGE biplot, interaction, organoleptic taste

## Abstract

**Introduction:**

Amaranth (*Amaranthus cruentus* L.) is an important leafy vegetable crop in sub-Saharan Africa, yet limited studies integrate genotype × environment (G × E) interaction analysis with gender-disaggregated participatory selection to guide breeding decisions.

**Methods:**

This study evaluated vegetable yield performance, agronomic traits, G × E interaction, and gender-disaggregated farmer preference of 23 *A. cruentus* genotypes, comprising 21 advanced breeding lines and two commercial varieties, under contrasting on-station and on-farm conditions in northern Tanzania. Trials were conducted using a randomized complete block design with three replications per location.

**Results:**

Significant G × E interactions were observed for marketable vegetable yield, biomass, and morphological traits. Mean marketable yield was higher at the World Vegetable Center Eastern and Southern Africa (WorldVeg-ESA) on-station location (37.75 t/ha) than at the Himo on-farm location (31.18 t/ha). GGE biplot analysis showed that PC1 and PC2 explained 81% and 19% of the total variation, respectively. Participatory evaluation showed overlapping preferences among male and female farmers, favoring genotypes with high biomass, narrow leaves, and fast and high regrowth ability.

**Discussion:**

Three promising genotypes were identified: AVAM2402 adapted to both locations, AVAM2404 adapted to Himo, and AVAM2408 adapted to WorldVeg-ESA. These lines will be advanced for distinctiveness, uniformity, and stability (DUS) testing for possible release as commercial varieties.

## 1 Introduction

Amaranth (*Amaranthus* spp.) is an important leafy vegetable and grain crop valued for its adaptation to diverse agroecological zones, and its nutritional value (Akinola, 2021). In Tanzania and other parts of sub-Saharan Africa, amaranth serves as a source of protein, vitamins A and C, calcium, iron, and zinc (Dinssa et al., 2016, 2019). The most widely grown species are *Amaranthus curentus* and *A. hypochondriacus* for both vegetables and grain, and *A. dubius* for vegetable production. Despite its nutritional and economic significance, amaranth production remains constrained by the limited availability of improved varieties adapted to local growing conditions, genotype-environment interaction (G x E), and suboptimal soil conditions (Cooper et al., 2022). Understanding genotype performance across different environments is therefore critical for enhancing yield, resilience, and adoption of amaranth varieties (Yadav and Yadav, 2024).

Factors such as soil fertility (including phosphorus content, pH value, and organic carbon concentration), along with moisture retention, and local temperature, collectively influence the performances of amaranth (De and Gomes, 2023). Azon et al. (2023) reported significant G × E interactions, showing that certain genotypes perform outstandingly under specific conditions but fail to perform optimally in others. Irrigation methods have also been shown to influence root development, water use efficiency and yield variability (Whitten, 2022). Only lines developed from genebank accessions were evaluated so far (Dinssa et al., 2019, 2022b), while the breeding lines from crossing program remain to be evaluated extensively.

Gender-disaggregated farmer participatory variety selection aligns cultivars with the demands set by the end users (Mengistu et al., 2025). Given that women play a dominant role in the production and marketing of traditional vegetables such as amaranth, their involvement is essential in evaluating field performance, organoleptic qualities (e.g., cooking and taste attributes), yield, nutritional value, and other plant traits to enhance adoption rates (Dinssa et al, 2022b; Whitson, 2010). Although previous studies have evaluated amaranth genotypes using either genotype × environment (G × E) analysis or participatory variety selection independently, limited research has systematically integrated gender-disaggregated participatory evaluation with formal G × E analysis of advanced breeding lines: these approaches have not yet translated into context-driven strategies that support the development of locally adapted, regionally appropriate, and high performing varieties (Dinssa et al., 2022b).

The present study was therefore conducted with the objective of evaluating 21 amaranth breeding lines, developed through a crossing program, along with two commercial varieties, to determine their adaptation, yield, and farmer acceptability. The findings will be used to guide breeding plans and guarantee that future varieties meet the diverse needs of stakeholders, ultimately contributing to improved nutrition security and livelihood in northern Tanzania and similar agro-ecological regions.

## 2 Materials and methods

### 2.1 Genetic materials

The genetic materials consisted of 23 A*. cruentus* entries, including 21 advanced breeding lines, F11 generation that are considered homozygous and genetically stable, developed by the World Vegetable Center Eastern and Southern Africa (hereafter WorldVeg-ESA) and two commercial varieties, Madiira 1 and Akeri (**Table 1**). The breeding lines were derived from a cross between ‘Madiira 1’ (female parent), known for its superior cooking and market qualities, but slow in seedling establishment, late in maturity, and low in seed yield to be accepted by the seed companies, and BRESIL(B)-Sel (male parent), a line recognized for its early growth vigor, early maturity, large 1000 grain weight, and high grain yield.

**Table 1:** Twenty-one amaranth lines and two varieties evaluated for marketable vegetable yield and organoleptic taste following farmers participatory approach in Tanzania in 2024^*^.

Genotype number	Genotype ame	Selection history	Growth habit	Stem pigmentation	Leaf pigmentation	Leaf shape	Leaf margin	Petiole pigmentation	Inflorescence colour	Stem colour
1	AVAM2401	CAMES15-22-1F1-10F2MR-6F3-28F4-7F5-4-5F6-1F7NMG-1F8-1-2F9-F10B1	Prostrate	Mixture	Normal green	elliptical	Entire	Mixture	Mixture	Gren
2	AVAM2402	CAMES15-22-1F1-10F2MR-31F3-108F4-153F5-22-8F6-14F7MR-13F8-5-2F9-F10B2	Prostrate	Purple or pink	Normal green	linear	Entire	Purple	Pink	Red
3	AVAM2403	CAMES15-22-1F1-10F2MR-46F3-152F4-236F5-40-2F6-20F7MR-23F8-7-2F9-F10B1	Prostrate	Purple or pink	Purple or pink	linear	Entire	Purple	Pink	Red
4	AVAM2404	CAMES15-22-1F1-10F2MR-49F3-167F4-253F5-51-2F6-22F7MG-25F8-8-1F9-F10B1	Prostrate	Green	Normal green	linear	Crenate	Green	Green	Green
5	AVAM2405	CAMES15-22-1F1-10F2MR-49F3-167F4-253F5-51-2F6-22F7MG-25F8-8-2F9-F10B1	Prostrate	Purple or pink	Normal green	linear	Entire	Green	Green	Green
6	AVAM2406	CAMES15-22-1F1-10F2MR-54F3-185F4-283F5-56-1F6-25F7MG-29F8-9-3F9-F10B1	Prostrate	Green	Normal green	linear	Entire	Green	Green	Green
7	AVAM2407	CAMES15-22-1F1-10F2MR-54F3-185F4-283F5-56-1F6-25F7MG-29F8-9-3F9-F10B2	Prostrate	Green	Normal green	linear	Entire	Green	Green	Green
8	AVAM2408	CAMES15-22-1F1-10F2MR-63F3-231F4-354F5-70-8F6-33F7NMG-42F8-11-3F9-F10B1	Prostrate	Purple or pink	Purple or pink	Lanceolate	Entire	Purple	Pink	Red
9	AVAM2409	CAMES15-22-1F1-10F2MR-63F3-231F4-355F5-70-9F6-34F7NMR-45F8-12-1F9-F10B1	Prostrate	Green	Normal green	elliptical	Entire	Green	Yellow	Green
10	AVAM2410	CAMES15-22-1F1-10F2MR-63F3-231F4-355F5-70-9F6-34F7NMR-45F8-12-1F9-F10B2	Prostrate	Green	Purple or pink	elliptical	Entire	Green	Pink	Green
11	AVAM2411	CAMES15-22-1F1-10F2MR-63F3-231F4-355F5-70-9F6-34F7NMR-45F8-12-1F9-F10B3	Prostrate	Green	Normal green	elliptical	Undulate	Green	Green	Green
12	AVAM2412	CAMES15-22-1F1-10F2MR-63F3-234F4-386F5-73-2F6-44F7NMR-57F8-14-3F9-F10B1	Prostrate	Purple or pink	Normal green	elliptical	Entire	Purple	Pink	Red
13	AVAM2413	CAMES15-22-1F1-10F2MR-63F3-234F4-389F5-73-5F6-47F7NMG-61F8-15-1F9-F10B1	Prostrate	Green	Normal green	elliptical	Entire	Green	Yellow	Gren
14	AVAM2414	CAMES15-22-1F1-10F2MR-63F3-234F4-389F5-73-5F6-47F7NMG-61F8-15-2F9-F10B1	Prostrate	Green	Normal green	Lanceolate	Entire	Green	Green	Green
15	AVAM2415	CAMES15-22-1F1-10F2MR-63F3-235F4-407F5-74-1F6-58F7NMG-80F8-18-2F9-F10B1	Prostrate	Green	Normal green	elliptical	Entire	Green	Green	Green
16	AVAM2416	CAMES15-22-1F1-10F2MR-64F3-242F4-465F5-81-1F6-66F7NMG-93F8-19-3F9-F10B1	Prostrate	Green	Normal green	elliptical	Entire	Green	Green	Green
17	AVAM2417	CAMES15-22-1F1-10F2MR-63F3-234F4-389F5-73-5F6-47F7NMG-61F8-15-1F9-40-1F10	Prostrate	Green	Normal green	elliptical	Entire	Green	Green	Green
18	AVAM2418	CAMES15-22-1F1-10F2MR-63F3-235F4-407F5-74-1F6-58F7NMG-80F8-18-1F9-50-1F10	Prostrate	Green	Normal green	elliptical	Entire	Green	Green	Green
19	AVAM2419	CAMES15-22-1F1-10F2MR-63F3-235F4-407F5-74-1F6-58F7NMG-80F8-18-3F9-52-1F10	Prostrate	Green	Normal green	elliptical	Entire	Green	Green	Green
20	AVAM2420	CAMES15-22-1F1-10F2MR-63F3-231F4-355F5-70-9F6-34F7NMR-45F8-12-2F9-33-1F10	Prostrate	Purple or pink	Normal green	Elliptical	Entire	Green	Green	Green
21	AVAM2421	CAMES15-22-1F1-10F2MR-63F3-235F4-405F5-74-3F6-56F7NMR-77F8-17-3F9-48-1F10	Prostrate	Purple or pink	Normal green	elliptical	Entire	Purple	Pink	Red
22	Madiira 1	EX-Zim-Sel	Erect	Green	Normal green	linear	Undulate	Green	Green	Green
23	Akeri	PARIS (A)-Sel	Prostrate	Purple or pink	Normal green	elliptical	Entire	Purple	Pink	Red

^*^The materials were characterized following the International Union for the Protection of New Varieties of Plants (2008) descriptors and/or descriptors used at the World Vegetable Center. Shanhua, Tainan. Taiwan.

### 2.2 Study location

The entries were evaluated in the open field at the WorldVeg-ESA research station in the Arusha region, and on-farm in Himo location, in the Kilimanjaro region, Tanzania. WorldVeg-ESA is located at a latitude of 3° 23’ 12.9300” S, longitude 36° 40’ 58.7820” E, and an elevation of 1235 m above sea level. The Himo location is located at a latitude of 3° 23’ 41” S, longitude 37°32’ 1” E, and an elevation of 870 m above sea level. The WorldVeg-ESA trial location in the Arusha region represented a cooler and humid environment, whereas the Himo on-farm location represented a low-altitude area in the Kilimanjaro region was hotter than the WorldVeg-ESA location.

**Figure 1:**
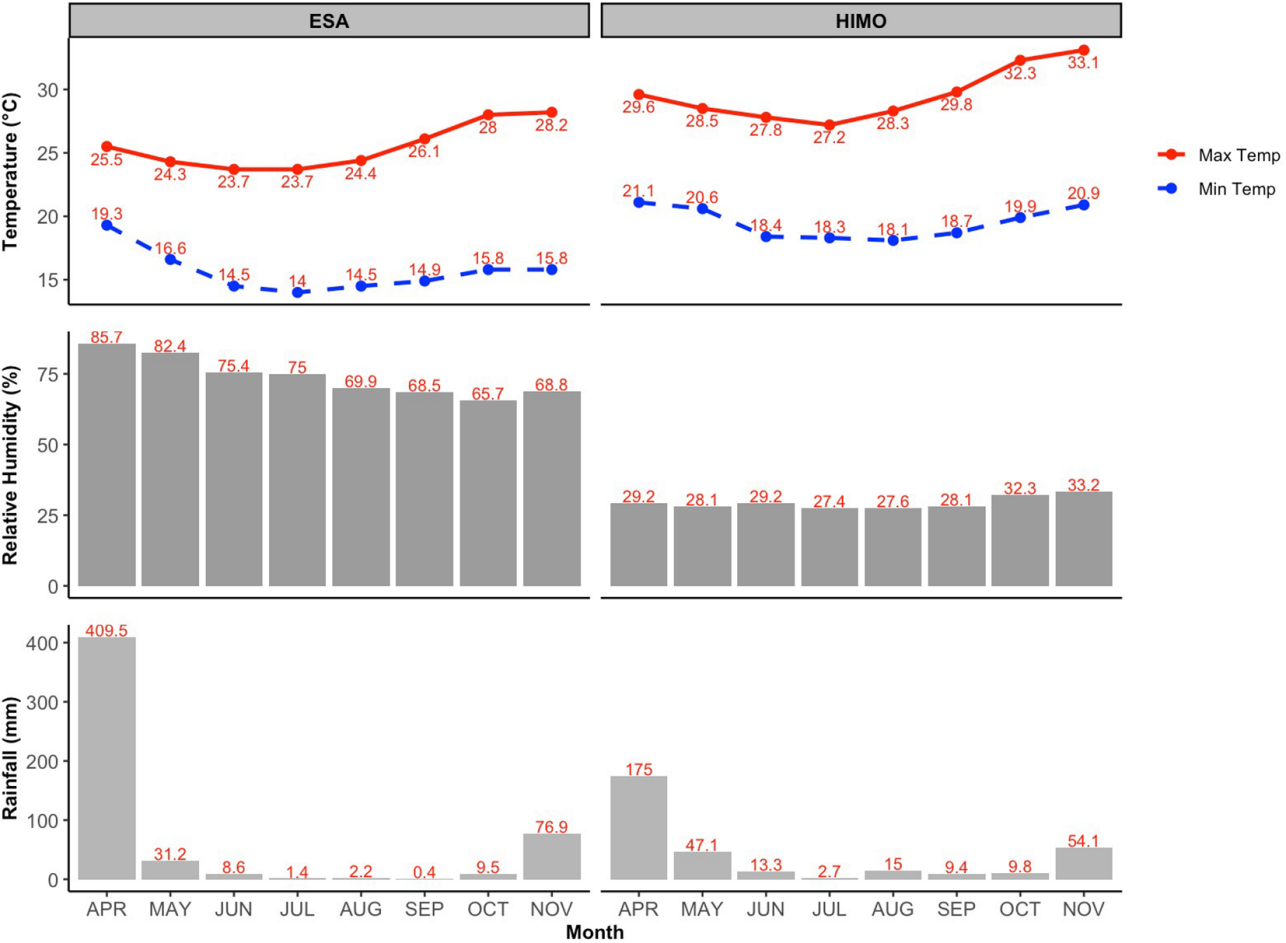
Mean minimum (Min Temp) and maximum (Max Temp) temperatures, relative humidity, and total rainfall (Rainfall) by month of World Vegetable Center Eastern and Southern Africa (ESA) in Arusha Region and that of Himo location in Kilimanjaro region during the trial season (April–November) in 2024.

Rainfall, temperature, and relative humidity data were sourced from the Tanzania Meteorological Authority. The mean minimum (Min) and maximum (Max) temperatures, relative humidity and total rainfall at the two locations are given in **Figure 1**. Soil samples were taken from the trial sites and analyzed at the Tanzania Agricultural Research Institute (TARI)-Mlingano. Primary soil samples were obtained from five spots using a Z-shaped sampling pattern across the trial field, at ploughing depth of 0–20 cm (Wang and Smith, 2004). Then the samples were combined and later reduced using the quartering method to obtain a representative sample weighing 0.5 kg (**Table 2**).

**Table 2:** Chemical and physical properties of soil (0–20 cm depth) at WorldVeg-ESA trial location in Arusha region and Himo location in Kilimanjaro region*.

Chemical property		WorldVeg-ESA	Himo
pH	(H2O)	5.70 ^MA^	6.20 ^SA^
pH	(Kcl)	5.10 ^MA^	5.40 ^MA^
EC	(mScm)	0.43 ^LS^	0.24 ^LS^
TN	(%)	0.02 ^VL^	0.02 ^VL^
OC	(%)	0.86 ^L^	1.29 ^M^
C:N		50.00 ^MQ^	66.00 ^MQ^
P	(mg/kg)	16.03 ^M^	3.33 ^VL^
K	(me/100g)	2.45 ^VH^	1.73 ^H^
Ca	(me/100g)	9.55 ^M^	5.35 ^M^
Mg	(me/100g)	4.54 ^H^	6.19 ^VH^
Ca: Mg		2.00 ^GQ^	1.00 ^GQ^
CEC	(me/100g)	32.36 ^H^	23.09 ^M^
BS	(%)	39.90 L	57.04 ^M^
Fe	(mg/kg)	52.76 ^VH^	52.00 ^VH^
Zn	(mg/kg)	3.93 ^H^	1.61 ^M^
Cu	(mg/kg)	0.86 ^VL^	0.78 ^VL^
Mn	(mg/kg)	17.42 ^H^	23.22 ^M^
Physical property			
%SAND	%	12	10
%SILT	%	30	26
%CLAY	%	58	64
Texture class		CLAY	CLAY

^*^Column 3 and 4: MQ, Moderate quality; GQ, Good quality; MA, Medium acid; SA, Slightly acid; LS, Low saline; VL, Very low; M, Medium; VH, Very high; H, High; L, Low. USDA texture interpretation manual, textural class triangle was used for texture.

At both locations, the experiment was conducted during a single growing season—the main vegetable growing season (May–October 2024), which corresponds to the cool dry season with intermittent rainfall. Seeds were sown in seedling trays on 07 May 2024 and were transplanted on 07 June at the WorldVeg-ESA on station. In Himo, sowing was on 15 July and transplanting on 05 August 2024.

### 2.3 Experimental design and field management

The study was laid out in a randomized complete block design (RCBD) with three replications at each location. Every genotype was transplanted in four rows with 11 plants each in a spacing of 60 cm between rows, and 30cm between plants. In both locations, basal NPK fertilizer (20N–4.4P–8.3K) was applied manually at the rate of 200 kg/ha one week after transplanting, followed by a side-dressing of urea (46N–0P–0K) at the rate of 120 kg/ha three weeks after transplanting. Drip irrigation was used at the WorldVeg-ESA, and furrow irrigation at the Himo Location.

### 2.4 Data collection

#### 2.4.1 Agronomic traits

In each location, plant height was measured at each harvest on three randomly sampled plants per plot, and the average of all harvests was taken as the plant height obtained under continuous or repeat harvest. Similarly, leaf length and leaf width were measured at each harvest on three fully developed leaves per plant (upper, middle and lower section of the plants) on each of three randomly sampled plants per plot. Plant height was measured from ground level to the apex of the plant. The number of branches per plant was counted at each harvest on three random plants per plot.

#### 2.4.2 Vegetable yield

Marketable vegetable yield per plot was determined by harvesting tender leaves and stems from 18 central plants from the two center rows of each plot. The plants were subjected to multiple harvests using a standard topping method. Harvesting was conducted by cutting one third of the main stem and branches measured from the apex, following the procedure used by Dinssa et al. (2019). The main stem height of each plant was measured from the ground to the apex, while the branch heights per plant were measured from the node on the main stem to the apex of the branch. The first harvest in each location was conducted 25-days after transplanting, with subsequent harvests carried out at 14-day intervals. A total of five harvests were conducted at each location. The cumulative yield of tender leaves and stems obtained from the five rounds of harvests per plot was used to determine marketable vegetable yield per plot.

#### 2.4.3 Root biomass and root length

Root biomass and root length were measured at the final harvest following completion of the fifth cutting cycle. Three representative plants per plot were carefully uprooted from the two central rows to avoid border effects. Roots were gently washed under running tap water to remove adhering soil particles. Root length was measured from the stem base to the tip of the longest root using a graduated ruler (cm). Fresh root biomass yield, after washing, was recorded using a digital weighing balance.

#### 2.4.4 Farmer participatory selection and organoleptic analysis

Farmer participatory variety selection was conducted at each location. At the WorldVeg-ESA site, 53 farmers participated (38 women and 15 men), while at the Himo site, 39 farmers took part (26 women and 13 men). To capture farmers’ preferences for the genotypes, a standardized 0–4 scoring scale was employed, where 0 = very poor, 1 = poor, 2 = good, 3 = very good, and 4 = excellent. Before the field plot evaluations, farmers held discussion with researchers to review the rating scales and agree on the traits to be considered when assigning scores. Through these discussions, farmers reached consensus to evaluate each plot based on visual observations, focusing on six key traits prioritized by both women and man: i) high biomass and rapid recovery after cutting, ii) marketability, iii) drought tolerance, iv) dark green leaf color, v) insect tolerance and disease resistance, and vi) ability for multiple harvesting. For statistical analysis, the selection scores for each plot were averaged across all participating farmers disaggregated by gender.

Organoleptic taste assessments were conducted at the Himo on-farm site under local farmer cooking conditions to capture consumer-relevant sensory preferences within a real production and consumption context. We focused on organoleptic taste evaluation at the on-farm location, as on-farm trials provide a broader representation of farmer-consumers and reflect local customary preparation practices more effectively than the on-station research setting. A total of 37 organoleptic taste evaluation panelist farmers (24 female and 13 male) were participated to identify their preferred genotype(s), using a 1–5 scale (1 = very poor taste, 2 = poor, 3 = good, 4 = very good, and 5 = excellent).

### 2.5 Data analysis

Statistical analyses were run in R software, version 4.4.2. The data homogeneity of variance and normal distribution to meet the assumptions of the analysis of variance (ANOVA) were tested. For biplot analysis, key packages included agricolae for statistical methods, ggplot2 for data visualization, and readxl for importing Excel datasets (Wickham et al., 2014). Combined analysis of variance (ANOVA) across the two locations was conducted using the model: Y_ijk_ = μ + G_i_ + L_j_ + (GL)_ij_ + B_k_(L_j_) + ϵ_ijk_, where μ is the overall mean; G_i_ represents the fixed effect of genotype; L_j_ represents the fixed effect of location; (GL)_ij_ represents the fixed genotype × location interaction; B_k_(L_j_) represents the random effect of replication nested within location; and ϵ_ijk_ represents the residual error term. Genotype and location were treated as fixed effects because the study aimed to compare specific genotypes across defined environments, whereas replication within location was treated as a random effect. Genotype and Genotype-by-Environment (GGE) interaction biplot analysis was conducted using environment-centered (location-centered) yield data to remove environmental main effects. Singular value decomposition (SVD) was applied to the G + GE matrix. Symmetric scaling (singular value partitioning = 2) was used so that genotype and environment scores were scaled proportionally. The first two principal components (PC1 and PC2) were retained to visualize genotype performance and stability across locations.

For analyzing marketable vegetable and related parameters, packages like dplyr were used for data manipulation and summarizing, alongside agricolae for least significant difference (LSD) analysis at a *p* = 0.05 significance level and ANOVA (Wickham et al., 2014). Moreover, for hierarchical clustering and dendrogram visualization, the dendextend package was applied with functions including dist(), hclust(), and color_branches(), enhancing the aesthetic and interpretation of dendrograms (Galili, 2015). Correlation analysis was conducted using R (Lüdecke et al., 2021) with the ggplot2 (Wickham et al., 2014) and ggcorrplot packages for visualization. Farmer selection scores (0–4 scale) were analyzed separately for each location using a two-way analysis of variance (ANOVA), with variety (23 levels) and gender (male, female) as fixed effects. The individual ANOVA on farmers’ selection scores was conducted separately for each gender group: the mean selection scores of all female farmers per plot were used for the analysis of female farmers’ selection scores, and the mean selection scores of all male farmers per plot were used for the analysis of male farmers’ scores. The interaction between variety and gender was tested. When significant differences were detected, mean separation was performed using LSD at P ≤ 0.05. Overall, this comprehensive integration of packages and functions enabled effective analysis and visualization of genotype-environment interactions.

## 3 Results

### 3.1 Soil

The soils at WorldVeg-ESA were moderately acidic (pH 5.7) and contained higher levels of phosphorus and potassium compared to those at Himo, which were slightly acidic (pH 6.2) and exhibited lower overall nutrient contents (**Table 2**).

The variation in marketable yield between WorldVeg-ESA and Himo occurred due to contrasting soil and environmental conditions between the two locations (**Table 2**). WorldVeg-ESA soil had higher available phosphorus (16.03 mg/kg) and exchangeable potassium (2.45 mg/100 g) compared with Himo (3.33 mg/kg P and 1.73 mg/100g). In contrast, Himo soils had greater clay content (64%) and higher organic carbon (1.29%) but lower nutrient availability.

However, because soil fertility, irrigation type, and climatic factors all covaried with site, it was not possible to disentangle soil effects from climatic influences in this experiment. Therefore, the observed yield differences should be interpreted as reflecting the combined environmental conditions of each location.

### 3.2 Agronomic traits

Plant height differed significantly among genotypes and between locations (P < 0.01) (**Table 3**). Mean plant height across sites ranged from 46.02 cm (AVAM2416) to 61.57 cm (AVAM2401). Plants were taller at WorldVeg-ESA (58.48 cm) than at Himo (47.35 cm). Genotypes AVAM2401, AVAM2408, AVAM2412, AVAM2414, AVAM2418, and Akeri exceeded 60 cm in at least one location.

**Table 3:** Agronomic traits (plant height, branch number per plant, leaf length, and leaf width) of 23 amaranth genotypes measured at five marketable vegetable yield harvest in trials conducted at WorldVeg-ESA (ESA) and Himo locations in Tanzania 2024*.

Genotype code	Plant height (cm)			Leaf Length (cm)			Leaf Width (cm)			Branches (no./plant)		
	ESA	Himo	Mean	ESA	Himo	Mean	ESA	Himo	Mean	ESA	Himo	Mean
AVAM2401	62.45	60.70	61.57	14.42	15.11	14.77	7.00	6.57	6.79	15.29	12.09	13.70
AVAM2402	58.63	47.46	53.05	17.05	16.19	16.62	5.44	4.50	4.97	15.07	13.93	14.50
AVAM2403	55.82	44.35	50.09	16.00	15.35	15.68	4.15	3.69	3.92	16.02	17.42	16.70
AVAM2404	54.05	47.94	51.00	17.12	18.02	17.57	4.22	3.88	4.05	16.80	15.16	16.00
AVAM2405	53.15	46.01	49.58	15.75	16.09	15.92	3.74	3.45	3.59	17.67	15.13	16.40
AVAM2406	56.37	42.96	49.66	14.38	15.09	14.74	3.87	3.61	3.74	19.93	14.49	17.20
AVAM2407	55.67	40.74	48.21	13.72	14.05	13.89	3.49	3.32	3.41	19.62	16.87	18.20
AVAM2408	62.90	47.31	55.11	11.67	12.15	11.91	5.74	5.37	5.55	18.53	18.18	18.40
AVAM2409	55.67	41.73	48.70	12.93	13.33	13.13	6.81	6.14	6.47	17.13	12.96	15.00
AVAM2410	57.19	54.93	56.06	14.13	15.27	14.70	7.30	7.33	7.31	13.58	11.22	12.40
AVAM2411	58.17	43.96	51.06	15.15	14.61	14.88	7.83	6.69	7.26	12.36	9.71	11.00
AVAM2412	64.52	57.30	60.91	13.51	13.76	13.64	8.02	7.04	7.53	13.13	9.56	11.30
AVAM2413	58.52	45.63	52.08	12.19	13.19	12.69	6.53	5.90	6.22	14.98	12.73	13.90
AVAM2414	63.20	44.32	53.76	13.63	13.47	13.55	7.43	6.44	6.94	14.64	14.67	14.70
AVAM2415	57.71	43.96	50.83	15.27	13.47	14.37	8.14	6.26	7.20	11.96	10.80	11.40
AVAM2416	50.87	41.16	46.02	12.64	11.92	12.28	5.57	4.74	5.15	15.22	12.18	13.70
AVAM2417	57.97	39.99	48.98	14.43	13.11	13.77	8.23	6.61	7.42	12.38	10.04	11.20
AVAM2418	63.91	40.26	52.08	13.99	13.41	13.70	6.89	5.74	6.32	18.62	14.76	16.70
AVAM2419	60.97	46.74	53.86	14.30	12.94	13.62	7.33	5.65	6.49	16.76	14.09	15.40
AVAM2420	59.01	52.17	55.59	14.41	14.62	14.51	8.01	6.99	7.50	13.00	10.47	11.70
AVAM2421	60.22	56.78	58.50	16.13	16.93	16.53	8.11	7.21	7.66	12.51	10.73	11.60
Madiira 1	56.86	44.68	50.77	15.89	15.66	15.77	3.53	3.15	3.34	18.82	17.78	18.30
Akeri	61.30	58.04	59.67	15.33	15.20	15.26	8.63	7.31	7.97	12.62	10.96	11.80
Means	58.48	47.35	52.92	14.52	14.48	14.50	6.35	5.55	5.95	15.51	13.30	14.40
F test (P)	0.00863	<0.001	<0.001	<0.001	<0.001	<0.001	<0.001	<0.001	<0.001	<0.001	<0.001	0.83
LSD0.05y	7.22	4.59	4.28	0.72	0.65	0.48	0.38	0.31	0.31	4.00	2.69	NS

^*^ESA, World Vegetable Center Eastern and Southern Africa, Arusha region.

^y^, NS, Non significance.

Leaf length also varied significantly among genotypes and between locations (P < 0.001). Mean values across locations ranged from 11.91 cm (AVAM2408) and 17.57 cm (AVAM2404).

The overall mean leaf length was 14.50 cm. Leaf width showed significant differences (P < 0.001) among genotypes. The narrowest leaves were recorded in Madiira 1 (3.34 cm) and AVAM2407 (3.41 cm), while the widest leaves were found in Akeri (7.97 cm) and AVAM2421 (7.66 cm). The overall mean leaf width was 5.95 cm (**Table 3**).

The number of branches per plant showed strong differences among genotypes (P < 0.001), but the combined analysis indicated no significant genotype × environment interaction (P = 0.830). Mean branch number ranged from 11.00 (AVAM2411) to 18.40 (AVAM2408), with Madiira 1 (18.30) and AVAM2408 (18.40) ranking among the highest. Branch number was higher at WorldVeg-ESA (15.51) than at Himo (13.30) (**Table 3**).

### 3.3 Marketable vegetable yield and biomass

Individual ANOVA indicated significant differences among the genotypes at the WorldVeg-ESA and Himo locations for marketable vegetable yield (**Table 4**). At the WorldVeg-ESA location, the marketable vegetable yield of the genotype ranged from 27.54 t/ha (AVAM2413) to 45.82 t/ha (AVAM2408), and at the Himo location, from 17.15 t/ha (AVAM2413) to 51.28 t/ha (AVAM2404). Combined analysis of variance in marketable vegetable yield indicated highly significant G x E interaction, and yields of the genotypes across locations ranged from 22.34 t/ha (AVAM2413) to 43.88 t/ha (AVAM2404) (**Table 4**). The individual ANOVA results indicated significant differences among the genotypes in aboveground biomass yield at the WorldVeg-ESA location and root biomass yield at Himo (**Table 4**).

**Table 4:** Fresh marketable vegetable yield, fresh above-ground biomass yield, root biomass and root length of 23 amaranth genotypes evaluated at WorldVeg-ESA (ESA) and Himo locations in Tanzania in 2024*.

Genotype	Marketable vegetable yield (t/ha)			Above-ground biomass (t/ha)			Root biomass (t/ha)			Root length (cm)		
	ESA	Himo	Mean	ESA	Himo	Mean	ESA	Himo	Mean	ESA	Himo	Mean
AVAM2401	37.78	37.35	37.57	60.26	53.56	56.91	3.68	3.94	3.81	25.62	29.47	27.54
AVAM2402	42.38	39.38	40.88	59.92	51.48	55.70	3.55	4.03	3.79	20.32	27.12	23.72
AVAM2403	37.65	36.69	37.17	59.51	53.56	56.54	3.36	3.80	3.58	22.78	27.77	25.27
AVAM2404	36.48	51.28	43.88	61.37	64.15	62.76	2.41	5.83	4.12	22.07	34.81	28.44
AVAM2405	37.45	43.76	40.60	59.52	57.89	58.70	2.62	3.75	3.18	25.04	27.88	26.46
AVAM2406	37.96	27.37	32.66	54.52	46.70	50.61	2.70	3.66	3.18	21.32	26.34	23.83
AVAM2407	37.00	23.13	30.06	55.00	37.74	46.37	3.67	2.69	3.18	24.08	24.73	24.41
AVAM2408	45.82	31.20	38.51	66.09	49.20	57.65	3.77	3.80	3.78	21.62	26.60	24.11
AVAM2409	35.98	22.35	29.16	62.42	42.07	52.25	3.25	4.17	3.71	21.52	26.63	24.08
AVAM2410	33.91	33.54	33.73	55.10	51.34	53.22	2.28	5.93	4.10	21.08	28.56	24.82
AVAM2411	36.61	26.65	31.63	54.31	32.69	43.50	2.81	4.63	3.72	21.39	26.26	23.82
AVAM2412	38.10	28.30	33.20	57.29	45.68	51.48	3.70	4.95	4.33	21.42	27.09	24.25
AVAM2413	27.54	17.15	22.34	45.44	33.88	39.66	3.20	4.49	3.84	23.74	29.34	26.54
AVAM2414	43.13	30.63	36.88	59.18	43.75	51.46	4.04	4.40	4.22	23.71	28.96	26.33
AVAM2415	37.04	26.90	31.97	55.05	36.18	45.61	3.19	5.56	4.38	22.69	29.90	26.30
AVAM2416	34.59	38.41	36.50	49.40	40.14	44.77	2.47	3.06	2.76	21.33	26.55	23.94
AVAM2417	32.83	21.51	27.17	45.89	30.51	38.20	5.17	4.26	4.72	26.58	28.15	27.37
AVAM2418	43.33	29.55	36.44	63.40	40.61	52.00	4.71	3.56	4.14	23.87	27.64	25.75
AVAM2419	35.51	29.64	32.57	54.75	41.98	48.37	4.33	3.10	3.72	23.36	28.34	25.85
AVAM2420	37.01	33.04	35.02	53.37	48.62	51.00	3.68	4.40	4.04	25.30	27.34	26.32
AVAM2421	39.60	26.18	32.89	58.68	47.47	53.08	3.50	3.98	3.74	23.91	28.67	26.29
Madiira 1	42.42	30.36	36.39	60.58	43.69	52.13	3.20	2.96	3.08	17.55	28.37	22.96
Akeri	38.05	32.77	35.41	58.11	51.75	54.93	3.57	4.40	3.99	22.08	29.02	25.55
Mean	37.75	31.18	34.46	56.92	45.42	51.19	3.43	4.14	3.79	22.71	28.07	25.39
F test (P)	<0.001	<0.001	<0.001	0.0382	0.161	0.8103	0.0658	0.0015	<0.001	0.21	0.33	0.28
lsd0.05x	6.48	7.28	4.84	10.75	NS	NS	NS	1.45	1.05	NS	NS	NS

^*^ESA, World Vegetable Center Eastern and Southern Africa, Arusha region.

x, NS, Non significance.

The first four entries with the highest marketable vegetable yield, plant height, number of branches per plant, above-ground biomass, root biomass, and female and male farmers selection scores at each location are given in **Table 5**. AVAM2408 showed the highest above-ground biomass (66.09 t/ha) and marketable vegetable yield (45.82 t/ha) in ESA, while AMAV2404 led in Himo with 64.15 t/ha above-ground biomass and 51.28 t/ha marketable vegetable yield.

**Table 5:** Four best amaranth genotypes for vegetable yield, female farmer group and male farmer group and other traits selected in each location measured on 23 genotypes grown in two locations Arusha and Kilimanjaro, Tanzania in 2024^y^,^z^.

Biomass above ground (t/ha)		Root Biomass (t/ha)		Plant height (cm)		Branches (no./plant)		Marketable vegetable yield (t/ha)		Female farmers selection score (0-4)		Male farmers selection score (0-4)	
Genotype code	Means	Genotype code	Means	Genotype code	Means	Genotype code	Means	Genotype code	Means	Genotype	Score (0-4)	Genotype	Score (0-4)
ESA	56.92	ESA	3.43	ESA	58.48	ESA	15.51	ESA	37.75	ESA	2.487	ESA	2.608
AVAM2408	66.09	AVAM2417	5.17	AVAM2412	64.52	AVAM2406	19.93	AVAM2408	45.82	Madiira 1	3.395	AVAM2404	3.111
AVAM2418	63.40	AVAM2418	4.71	AVAM2418	63.91	AVAM2407	19.62	AVAM2418	43.33	AVAM2405	3.316	Madiira 1	3.044
AVAM2409	62.42	AVAM2419	4.33	AVAM2414	63.20	Madiira 1	18.82	AVAM2414	43.13	AVAM2404	3.298	AVAM2402	3.00
AVAM2404	61.37	AVAM2414	4.04	AVAM2408	62.90	AVAM2418	18.62	Madiira 1	42.42	AVAM2403	3.272	AVAM2405	2.911
Himo	45.42	Himo	4.14	Himo	47.35	Himo	13.30	Himo	31.18	Himo	2.094	Himo	2.32
AVAM2404	64.15	AVAM2410	5.93	AVAM2401	60.70	AVAM2408	18.18	AVAM2404	51.28	AVAM2404	2.923	AVAM2410	2.897
AVAM2405	57.89	AVAM2404	5.83	Akeri	58.04	Madiira 1	17.78	AVAM2405	43.76	AVAM2405	2.859	AVAM2404	2.872
AVAM2401	53.56	AVAM2415	5.56	AVAM2412	57.30	AVAM2403	17.42	AVAM2402	39.38	AVAM2417	2.462	AVAM2405	2.692
AVAM2403	53.56	AVAM2412	4.95	AVAM2421	56.78	AVAM2407	16.87	AVAM2416	38.41	AVAM2410	2.449	AVAM2417	2.564

^y^, ESA, World Vegetable Center Eastern and Southern Africa, Arusha region.

^z^, 0, very poor; 4, excellent.

Yield performance corresponded with temperature and relative humidity differences, cooler and more humid environment of Worldveg-ESA favored vegetative growth, while the hotter and drier conditions of Himo limited biomass accumulation.

### 3.4 Farmer participatory evaluation and organoleptic taste

Individual ANOVA indicated significant differences among entries in female farmers’ selection scores at Himo, and in female and male farmers selection scores at the WorldVeg-ESA (**Table 6**). Combined ANOVA of female plus male farmers’ selection scores indicated significant differences among entries at WorldVeg-ESA location, while non-significant differences among genotypes at the Himo location. The mean female farmers’ selection score ranged from 1.75 to 3.40 at the WorldVeg-ESA, 1.39 to 2.92 at Himo. Male farmers’ selection score ranged from 2.18 to 3.11 at the WorldVeg-ESA, while 1.67 to 2.90 at Himo.

**Table 6:** Gender-disaggregated farmers’ selection scores of 23 amaranth genotypes evaluated at WorldVeg-ESA (ESA) and Himo locations, and taste panel scores on samples harvested from Himo location trial in Tanzania in 2024^v^.

	Himo			ESA			Proportion of panelists who gave different taste panel scores on the scale of 1-5 (%)y		
	Female farmers selection score (0-4)	Male farmers selection score (0-4)	Mean	Female farmers selection score (0-4)	Male farmers selection score (0-4)	Mean			
Genotype code	(n=26)	(n=13)	(n=39)	(n=38)	(n=15)	(n=53)	Panelist(no.)	Score ≥ 3	Score ≥ 4
AVAM2401	2.05	2.41	2.69	2.13	2.51	2.32	37	73.00	37.80
AVAM2402	1.78	2.03	3.00	3.18	3.00	3.09	37	70.30	45.90
AVAM2403	2.32	2.44	2.77	3.27	2.91	3.09	37	59.50	40.50
AVAM2404	2.92	2.87	3.39	3.30	3.11	3.21	37	75.70	48.60
AVAM2405	2.86	2.69	3.15	3.32	2.91	3.11	37	70.30	45.90
AVAM2406	2.37	2.41	3.15	2.77	2.49	2.63	37	73.00	37.80
AVAM2407	2.30	2.03	2.58	2.68	2.62	2.65	37	70.30	37.80
AVAM2408	1.39	1.74	2.00	2.24	2.38	2.31	37	73.00	56.80
AVAM2409	2.22	2.54	2.92	2.22	2.47	2.34	37	59.50	45.90
AVAM2410	2.45	2.90	3.08	2.22	2.56	2.39	37	51.40	37.80
AVAM2411	2.03	2.28	2.69	2.10	2.51	2.30	37	73.00	43.20
AVAM2412	1.83	2.36	2.62	2.32	2.42	2.37	37	73.00	37.80
AVAM2413	1.67	2.26	2.54	2.13	2.42	2.28	37	75.70	62.20
AVAM2414	1.95	2.51	3.23	2.47	2.87	2.67	37	75.70	37.80
AVAM2415	2.05	2.28	3.08	2.13	2.53	2.33	37	64.90	45.90
AVAM2416	1.58	1.67	1.85	1.75	2.18	1.96	37	78.40	54.10
AVAM2417	2.01	2.26	2.46	2.40	2.82	2.61	37	56.80	37.80
AVAM2418	1.90	2.13	2.54	2.05	2.29	2.17	37	48.60	29.70
AVAM2419	2.06	2.13	2.62	2.43	2.64	2.54	37	81.10	48.60
AVAM2420	2.46	2.56	2.77	2.38	2.67	2.52	37	70.30	40.50
AVAM2421	1.81	2.33	2.62	2.16	2.33	2.25	37	75.70	51.40
Madiira 1	2.30	2.44	2.85	3.4	3.04	3.22	37	51.40	27.00
Akeri	1.86	2.10	2.54	2.18	2.29	2.24	37	56.80	43.20
Mean	2.10	2.32	2.21	2.49	2.61	2.55	NA	NA	NA
F test (P)	<0.001	0.138	0.9687	<0.001	0.00139	0.0895	NA	NA	NA
lsd0.05x	0.56	NS	NS	0.34	0.45	NS	NA	NA	NA

^v^, ESA, World Vegetable Center Eastern and Southern Africa, Arusha region.

^w^, 0–4 scoring scale 0 = very poor, and 4 = excellent.

^x^, numbers in brackets stand for number of female and male farmers participated in the selection.

^y^, organoleptic taste score 1 = very poor, not liked by the panelist; 5 = excellent, highly liked.

^z^, NS, non-significant; NA, not applicable.

The organoleptic taste revealed clear genotype differences in consumer preference (**Table 6**). At least 65% of the 23 genotypes evaluated received ≥3 organoleptic taste score (on a 1–5 scoring scale) from at least 70% of the 37 panelists. Genotypes AVAM2413, AVAM2408, AVAM2416 and AVAM2421, in that order, received the highest mean taste scores (≥4.0) from at least 51% of the panelists for their overall taste and leaf tenderness. ‘Madiira 1’, the commercial variety was among the least preferred genotypes in the organoleptic evaluation.

### 3.5 GGE (genotype and genotype × environment) biplot

The GGE biplot illustrates the performance of the genotypes in marketable vegetable yield across the two locations, WorldVeg-ESA and Himo, by using two principal component axes (PCs) (**Figure 1**). PC 1 accounted for 81% of the total variation, reflecting the primary differences in genotypic performance, while PC 2 captured an additional 19%. Given that only two environments were included, the high proportion of variance explained by PC1 was expected. Each point on the plot represents a specific amaranth genotype, with its position indicating how well it performs in relation to the locations. Genotypes such as ‘Akeri’ (code 23) closer to the center line highlight adaptability to both locations, but not necessarily at high yield level. On the other hand, AVAM2404 (4) and AVAM2405 (5) clustering together were better adapted to Himo, indicating adaptation to a specific location, with similar performance in terms of marketable vegetable yield. In contrast, genotypes further from the environment markers (WorldVeg-ESA and Himo), for instance AVAM2413 (13), had lower adaptability or inconsistent yields. Genotypes located near the origin of the biplot exhibited greater stability, whereas those further along PC1 showed higher mean yield but site-specific adaptation. Overall, the biplot served as an insightful tool for identifying superior genotypes with narrow and broader adaptation, guiding decisions on which varieties to recommend for which environment.

### 3.6 Dendrogram

**Figure 2:**
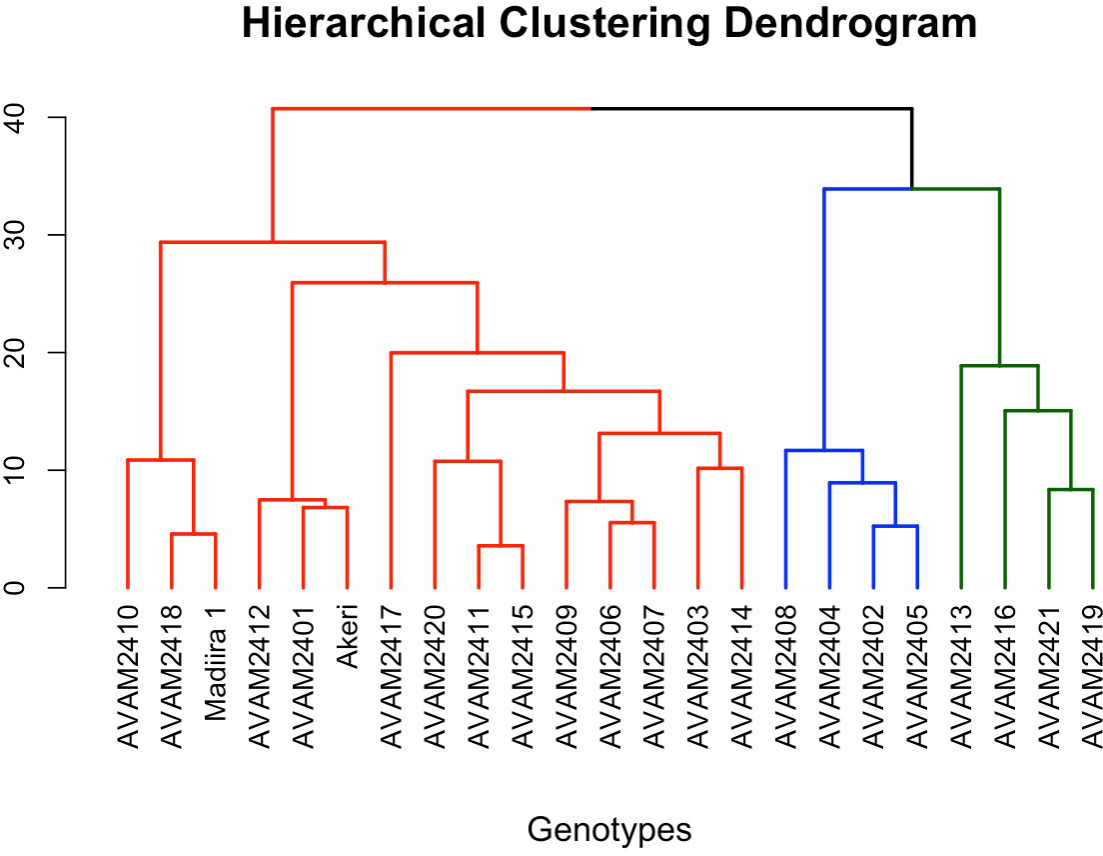
Dendrogram cluster of 23 genotypes for marketable vegetable yield, agronomic trait, organoleptic taste, farmer selection score and biomass 2024.

Hierarchical clustering (dendrogram) was performed using Euclidean distance and Ward’s linkage method based on standardized trait data on marketable vegetable yield, plant height, leaf length, leaf width, number of branches per plant, taste score, farmer selection scores, above-ground biomass, and root biomass (**Figure 2**). The genotypes were classified into three major clusters. Cluster one included the majority (15) of the genotypes with moderate average values across all measured traits, particularly for marketable yield (33.6 t/ha), aboveground biomass (50.41 t/ha), and farmer selection scores (2.66). These genotypes had the lowest consumer preference (51.26%) (**Figure 2**). Cluster two contained four genotypes with high values across all measured traits, particularly for marketable vegetable yield (average 40.97 t/ha), farmer selection score (2.91), and above-ground biomass (58.70 t/ha), and moderate taste scores (60.81%). Cluster three, also with four genotypes, had the lowest average marketable yield (31.08 t/ha), aboveground biomass yield (46.47 t/ha), and farmer selection score (2.33), however, showed the highest taste scores (65.90%), indicating that these genotypes have some market value, despite their lower productivity.

### 3.7 Correlation analysis

**Figure 3:**
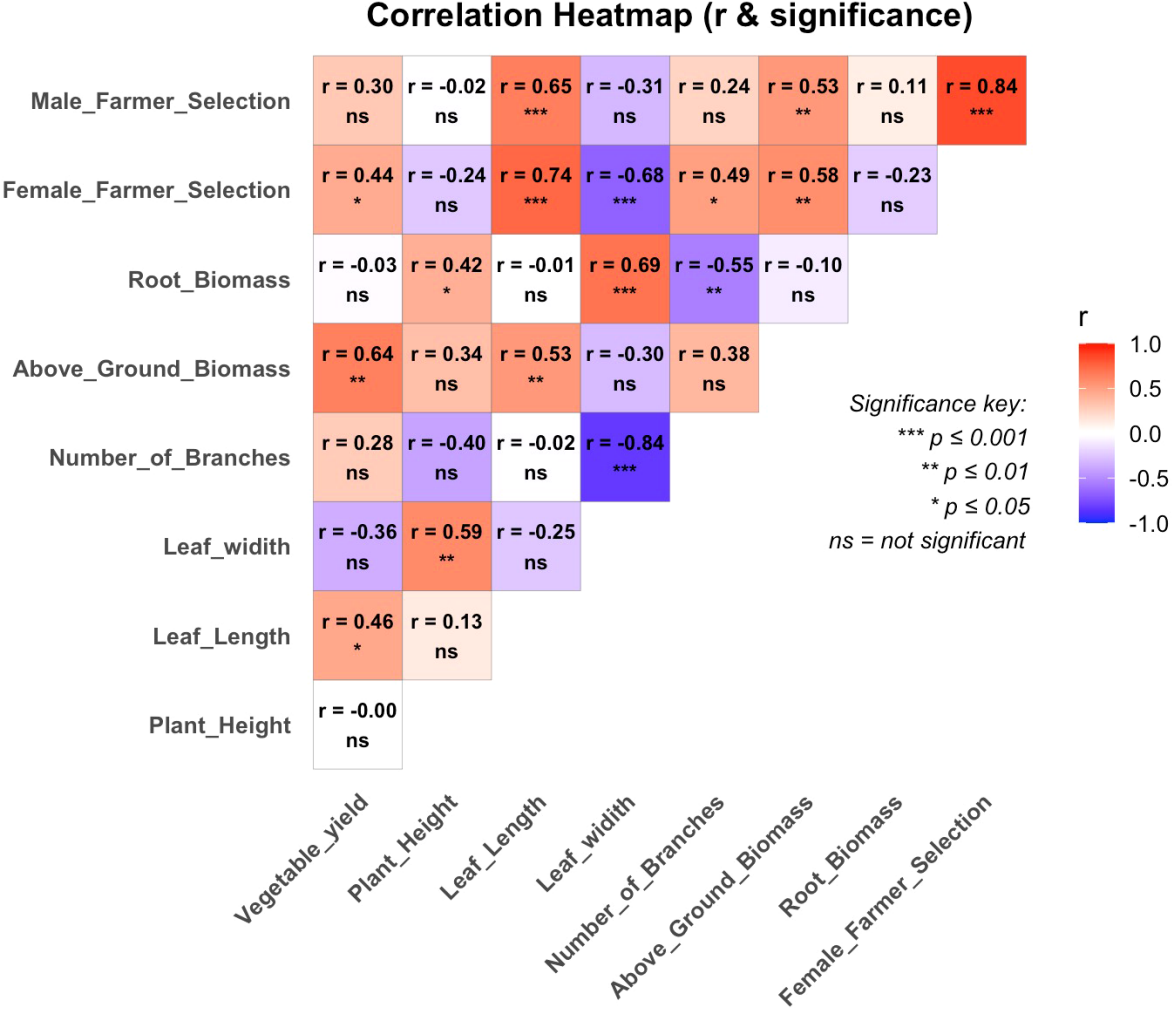
Correlation for marketable vegetable yield, agronomic trait, farmer selection score, and biomass yield 2024. *, ** and *** significant at p < 0.05, p < 0.01 and p < 0.001, respectively, and ns = nonsignificant.

Correlation analysis showed that male and female farmers gave higher preference scores to high-yielding genotypes. Above-ground biomass was associated with marketable vegetable yield (**Figure 3**). Leaf length had a significant positive correlation with yield (r = 0.46*). Plant height was not significantly correlated with yield but showed positive correlation with leaf width (r = 0.59**). Root biomass was not significantly correlated with marketable vegetable yield, but it showed a highly significant positive correlation with leaf width (r = 0.69**) (**Figure 3**).

## 4 Discussion

**Figure 4:**
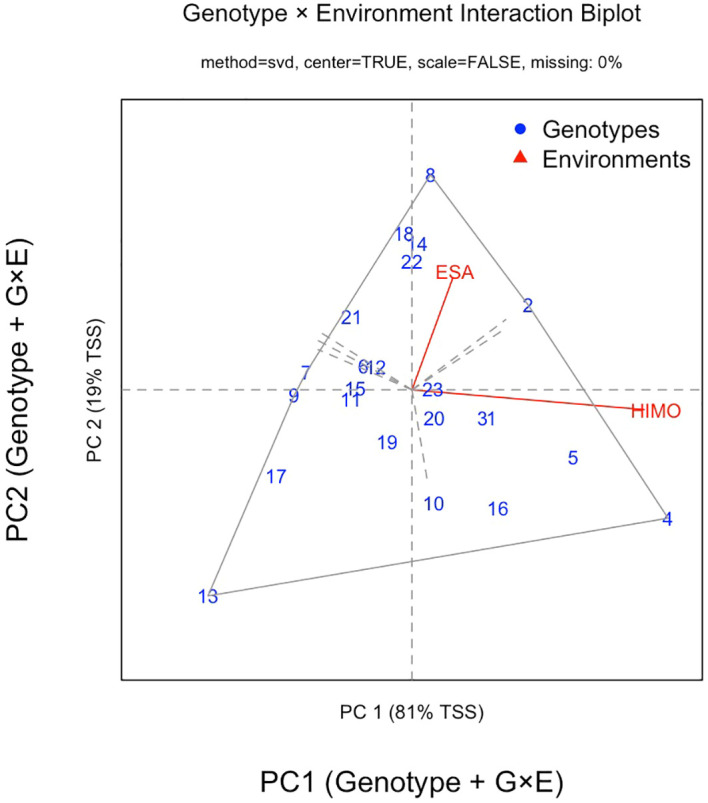
GGE biplot for marketable vegetable yield of 23 amaranth genotypes grown at World Vegetable Center Eastern and Southern Africa (ESA) in Arusha region and at Himo location in Kilimanjaro region in Tanzania in 2024. Pink line stands for locations: ESA and Himo.

In this study, 23 diverse amaranth genotypes were evaluated in replicated trials to assess their performance in terms of vegetable yield, farmer preferences, taste preference, and other traits across two contrasting environments in northern Tanzania. The locations differed in altitude, soil properties and weather conditions. The results demonstrated strong environmental influence on genotype performance. The GGE biplot grouped the two environments into different cluster groups based on marketable yield (**Figure 4**), supporting previous findings that amaranth yield is environment-dependent (Dinssa et al., 2019).

ANOVA highlighted significant differences in marketable yield and agronomic traits of the genotypes between WorldVeg-ESA and Himo locations (**Tables 3**, **4**). Because soil properties, irrigation practices, and climatic variables co-varied with location, it was not possible to isolate the effect of individual environmental factors. Therefore, yield differences should be interpreted as reflecting combined site-specific environmental conditions rather than the effect of a single factor. WorldVeg-ESA location produced higher average marketable yield than Himo, likely reflecting its more favorable soil nutrient profile and higher relative humidity that supported vegetative growth. The wider yield range observed at Himo suggests that the warmer and lower humidity conditions accentuated genotypic differences, revealing adaptive variation among genotypes. Soil parameters can be regarded as primary determinants of biomass production, with marketable yield responding secondarily through biomass accumulation. Irrigation was used in the present study to have optimum soil moisture for plant growth. Most farmers in Tanzania and other African countries, however, lack irrigation facilities, hindering vegetable production during the dry season and require varieties that can cope with low moisture levels (Dinssa et al., 2019). The significant difference in marketable yield and plant height between the locations underscores the influence of environmental factors such as soil parameters, climate and type of irrigation method (Richiedei et al., 2024; Shango et al., 2021). Dinssa et al. (2019 and 2022b) reported higher marketable amaranth yield in locations characterized by high temperature and low altitude, which contrasts with the findings of the present study suggesting the involvement of additional interacting environmental factors such as soil nutrient contents. According to Oliveira et al. (2025), the significant differences among locations in yield and agronomic traits indicate the necessity for targeted breeding efforts that consider environmental influences, aiming to develop high yielding varieties suited to diverse growing conditions, and varieties adapted to specific environmental conditions thereby enhancing productivity and sustainability in amaranth cultivation.

In the present study, the combined ANOVA results revealed significant interaction effects (G × E) for marketable vegetable yield and agronomic traits such as plant height, leaf length and leaf width, indicating that the performance of the amaranth genotypes evaluated varied between WorldVeg-ESA and Himo environments. Specifically, some genotypes exhibited stable performance across both locations (widely adapted), whereas others demonstrated location-specific superiority (specifically adapted). The two locations differed in several environmental aspects, including soil nutrient status, temperature, humidity, and irrigation method (Anderson, 1970). For instance, available soil phosphorus was higher at WorldVeg-ESA (16.03 mg/kg) than at Himo (3.33 mg/kg) (**Table 2**) (Murphy, 2015). This substantial difference in phosphorus availability suggests that nutrient status, particularly phosphorus, may have contributed to the higher yields recorded at WorldVeg-ESA (Nziguheba et al., 2016). However, because multiple environmental variables co-varied with location, the experimental design does not permit isolation of individual environmental effects. Consequently, interpretation should be confined to the observed G × E interaction, which represents the combined influence of these environmental factors. The magnitude of G × E interaction observed here indicates that some genotypes expressed relatively stable yields across locations, whereas others exhibited strong location-specific adaptation, insights directly relevant for breeding and variety recommendation. By focusing on G × E interactions, breeders can select genotypes with high yield potential and resilience across diverse growing conditions, either prioritizing widely adapted genotypes for broad cultivation (Cooper and Messina, 2023) or specifically adapted genotypes for targeted environments. Ultimately, understanding G x E interaction effects is crucial for enhancing the adaptability and productivity of amaranth, ensuring that developed varieties are well suited to the varied environmental contexts in which they will be cultivated (Bybee-Finley, 2021). The focus on high yielding varieties will not only benefit farmers but also contribute to food security (Shiferaw et al., 2013), and nutrition security.

The root biomass measurements and root length data are important for understanding water and nutrient uptake capabilities (Wang and Smith, 2004). The longer root lengths observed at Himo may reflect differences in irrigation regime and soil conditions. Drip irrigation as used at WorldVeg-ESA results in shallower roots (Mahinda et al., 2016). Also, the soil pH (6.2) at Himo is more alkaline than the one at WorldVeg-ESA (5.4), which can influence root development (Pearson, 2015). The ANOVA results revealed significantly higher root biomass at Himo, suggesting that environmental factors, farrow irrigation, might played a pivotal role for this parameter (Lynch et al., 2011).

Gender-disaggregated participatory evaluation for preferred genotypes is critical in breeding programs, particularly for African traditional vegetables, where information on market preferences and product profiles required by different farmer and consumer groups remains limited (Dinssa et al., 2022a). Most of the traits prioritized by farmers in the current study were also reported to be of importance to farmers (Dinssa et al., 2020, 2022a, Adeniji and Aloyce, 2013). In the current study, the most important traits identified by both female and male farmers were high biomass and marketability, dark green leaf color, and ability to be harvested several times from the same planting. The strong positive correlation observed between male and female selection scores indicates substantial agreement in varietal preferences between the two gender groups. Genotypes consistently preferred across locations demonstrate both agronomic performance and farmer acceptance, strengthening their candidacy for dissemination across the locations. The ANOVA results revealed significant differences in selection scores among female farmers only at Himo and in both female and male farmers at WorldVeg-ESA, suggesting that the preferences of farmers in different location may differ. Dinssa et al. (2022a) and Voss et al. (2025) reported that developing varieties appealing to the needs of both women and men is critical. In agreement with the present study, Dinssa et al. (2022a) reported a positive correlation between marketable vegetable yield and both female and male farmers’ selection scores, as well as a positive and significant correlation between the selection scores of the two selector groups. The negative correlations of leaf width with farmers’ selection scores may suggest that narrow leaf type varieties are preferred and are associated with good cooking and taste quality traits liked by farmers.

The dendrogram depicting the trait diversity of the germplasm panel reveals three distinct clusters based on marketable yield, agronomic traits, preferred taste and biomass (**Figure 2**). Cluster II contained high-yielding and widely accepted genotypes suitable for commercial production across environments, while Cluster III grouped low yielding but high taste quality genotypes that may serve niche markets or function as quality donors in breeding programs. The clustering analysis highlighted phenotypic relationships among genotypes, thereby informing targeted breeding strategies that prioritize high yield and marketability, consistent with approaches described by Cobb et al. (2013), while also incorporating organoleptic taste quality traits. By identifying traits from genotypes in top performing clusters, breeders can develop improved varieties that enhance both productivity and consumer preferred traits (Tiwari et al., 2022).

The correlation analysis between marketable vegetable yield, farmer selection scores, and various agronomic traits offers valuable insights in factors influencing amaranth adoption (Dinssa et al., 2019; Dinssa, et al., 2022b). Previous studies by Dinssa et al. (2019, 2022b) reported a positive correlation between marketable vegetable yield and leaf length and leaf width during the dry season, and with number of branches per plant during the wet-cool season. In the current study, marketable vegetable yield showed significant positive correlation with leaf length, number of branches per plant, and highly significant positive correlation with above ground biomass. These findings indicate that above-ground biomass is a strong predictor of marketable vegetable yield, suggesting that selection for increased biomass could indirectly enhance marketable yield.

In summary, we evaluated 23 amaranths germplasm (21 breeding lines and two varieties) across two contrasting locations in northern Tanzania, WorldVeg-ESA on-station (cool and humid environment) and Himo on-farm (hotter and low humidity environment). The study revealed significant environmental influences on yield and agronomic traits, with WorldVeg-ESA location producing higher marketable yield (37.8 t/ha on average) as compared to Himo with the location average yield of 31.3 t/ha, likely reflecting differences in soil nutrient contents and weather conditions. Farmer preferences varied with location, favoring ‘Madiira 1’ in WorldVeg-ESA location and AVAM2404 in Himo location. Correlation analysis indicated association between above-ground biomass and marketable vegetable yield. Cluster analysis identified high performing genotypes in one cluster group facilitating their strategic use in breeding programs. To enhance productivity and adoption, breeding efforts should prioritize both widely adapted and specifically adapted genotypes that incorporate farmer preferred traits, while accounting for gender-based differences and similarities in preference. However, the findings of this study should be interpreted within the context of certain limitations. The evaluation was conducted during a single growing season, and therefore seasonal variability was not captured. In addition, the study did not include an economic analysis, which is not commonly conducted in breeding nurseries and trials, to assess the cost–benefit implications of the selected genotypes. Future research conducted across multiple seasons would provide stronger evidence to support the recommendation of varieties for dissemination across wider environments.

## Data Availability

The raw data supporting the conclusions of this article will be made available by the authors, without undue reservation.
